# Brain Monitoring: Do We Need a Hole? An Update on Invasive and Noninvasive Brain Monitoring Modalities

**DOI:** 10.1155/2014/795762

**Published:** 2014-01-23

**Authors:** D. G. Barone, M. Czosnyka

**Affiliations:** ^1^Department of Neurosurgery, The Walton Centre, Liverpool L9 7AL, UK; ^2^Department of Academic Neurosurgery, Addenbrooke's Hospital, University of Cambridge, P.O. Box 167, Cambridge CB2 0QQ, UK

## Abstract

The ability to measure reliably the changes in the physical and biochemical environment after a brain injury is of great value in the prevention, treatment, and understanding of the secondary injuries. Three categories of multimodal brain monitoring exist: direct signals which are monitored invasively; variables which may be monitored noninvasively; and variables describing brain pathophysiology which are not monitored directly but are calculated at the bedside by dedicated computer software. Intracranial pressure (ICP) monitoring, either as stand-alone value or study of a dynamic trend, has become an important diagnostic tool in the diagnosis and management of multiple neurological conditions. Attempts have been made to measure ICP non-invasively, but this is not a clinical reality yet. There is contrasting evidence that monitoring of ICP is associated with better outcome, and further RCTs based on management protocol are warranted. Computer bedside calculation of “secondary parameters” has shown to be potentially helpful, particularly in helping to optimize “CPP-guided therapy.” In this paper we describe the most popular invasive and non invasive monitoring modalities, with great attention to their clinical interpretation based on the current published evidence.

## 1. Introduction

Brain injury occurs either at the time of a direct insult or subsequently due to changes in the physical and biochemical environment. The ability to measure these changes reliably is of paramount importance in order to tailor the treatment to each individual patient and therefore prevent the onset of secondary brain injury.

Multimodal brain monitoring can be grouped into three categories:

1. *direct signals* which are *monitored invasively* (e.g., intracranial pressure (ICP), tissue oxygenation, microdialysis, parenchymal blood flow, etc.);
2. variables which may be monitored noninvasively (e.g., transcranial Doppler (TCD) or near infrared spectroscopy (NIRS));
3. *variables* describing brain pathophysiology which are not monitored directly but are calculated at the bedside *by dedicated computer software*. The simplest example is the cerebral perfusion pressure (CPP), which is the difference between the mean arterial blood pressure (MAP) and the ICP (CPP = MAP-ICP), and therefore it is a calculated variable. More sophisticated examples include various indices of vascular reactivity or cerebral autoregulation [1], brain compensatory reserve [2], vascular resistances, and brain compartmental compliances [3].

## 2. Intracranial Pressure (ICP)

Even though since the end of the 19th century the spinal CSF pressure was used as indirect measure of ICP, the first reports of the use of continuous intracranial pressure monitoring via ventricular catheter were by Guillaume and Janny in 1951 [4] and later by Lundberg in 1960 [5]. With time, ICP monitoring has become an important component in the diagnosis and management of multiple neurological conditions, such as head injury, hydrocephalus, subarachnoid haemorrhage, and intracranial haematoma.

The particular anatomy of the brain, which is enclosed and protected by a rigid skull, creates a unique pressure-volume relationship compared to the rest of the human body, as already described by Monroe and Kelly more than 200 years ago. In normal circumstances the ICP is kept in its normal range, maintaining the relationship between the cerebrospinal fluid (CSF), the intracerebral blood, and brain tissue constant. In current clinical practice the ICP is measured invasively using an intracranial (ventricular, parenchymal, subdural, or extradural) catheter connected to or integrated with a pressure transducer.

**Figure 1:**
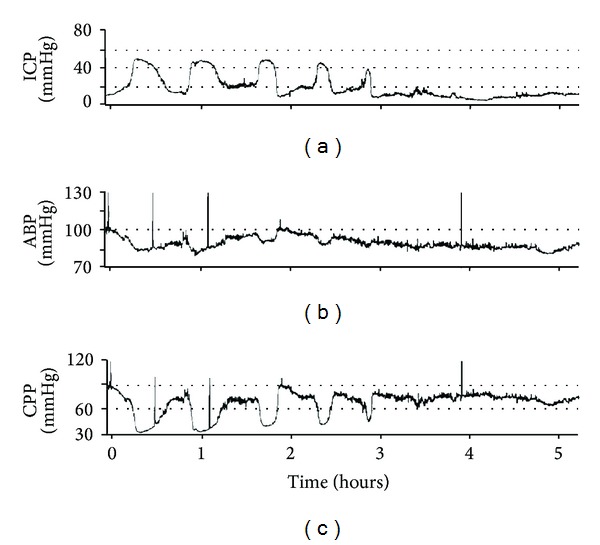
Case of a head injury patient with a highly unstable ICP, due to “plateau” waves. Details are in the text. ICP: intracranial pressure. ABP: arterial blood pressure. CPP: cerebral perfusion pressure.

The interpretation of ICP using the single “number,” either at one moment in time or the average over a period of time (e.g., daily ICP), although useful, can often be misleading.  Figure 1 demonstrates the case of a head injury patient with a highly unstable ICP, due to “plateau” waves. It has a dynamic trend, ranging from 10 to above 55 mmHg, and any increase in ICP follows a decrease in CPP down to 40 mmHg, during which secondary “brain insults” happen. Such high ICP dynamics is not always well represented by its average value and only continuous trending, with a good representation of monitoring values, can reveal such a hyperdynamic state.

**Figure 2:**
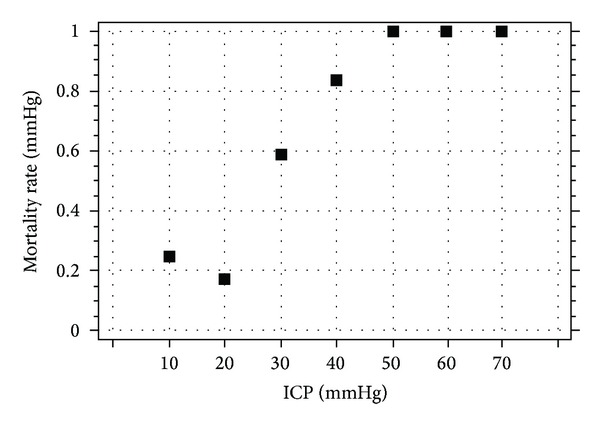
Correlation between mortality rate and ICP. General threshold between normal and elevated ICP between 20 and 30 mm Hg. ICP: intracranial pressure.

The single value has a good potential to support statistical studies. In the study from 1996, the distribution of mean ICP in different outcome groups reveals that those who died have much greater mean ICP than survivors [6]. Interestingly in survivors outcome groups there is no difference between averaged ICP between favourable and unfavourable outcomes. Mean ICP may be also used to establish general threshold between normal and elevated ICP (Figure 2). Undoubtedly, it is between 20 and 30 mmHg, where mortality rate starts to increase with ICP [7].

There is also evidence that thresholds may be found and traced in time for individual cases [8]. Index of cerebrospinal compensatory reserve, plotted against ICP, can show “saturation,” associated with exhausted reserve above a certain pressure. Above this level, any further rise of intracerebral volume may lead to refractory intracranial hypertension. This phenomenon is undoubtedly fatal. Rise in ICP may be fast. It is associated with decrease in CPP and cerebral ischaemia.

A recent RCT conducted by Chesnut and colleagues demonstrated that for patients with severe brain injury care that focused on maintaining monitored ICP at 20 mmHg or less was not shown to be superior to care based on imaging and clinical examination [9]. However a “normal” ICP should not be considered only in light of a particular cut-off value, because waveform analysis of the ICP is also important. ICP waveform analysis can provide information on the state of cerebrovascular reactivity (PRx) and can be used to estimate optimal cerebral perfusion pressure levels for individual patients as described later in this paper [10].

## 3. Pressure Reactivity Index (PRx)

**Figure 3:**
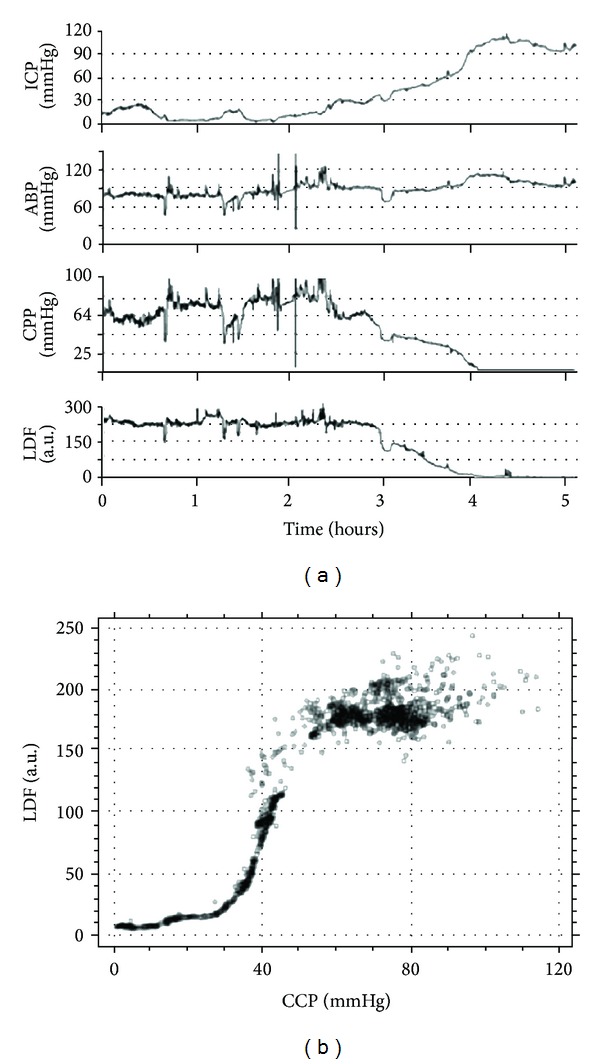
(a) Cortical cerebral blood flow (CBF) can be monitored with laser Doppler flowmetry, and CBF plotted against CPP shows the autoregulatory curve, called Lassen's curve, with clear lower limit of autoregulation (LLA). ICP: intracranial pressure. ABP: arterial blood pressure. CPP: cerebral perfusion pressure. LDF: laser Doppler flowmetry. (b) Lassen's curve. CPP: cerebral perfusion pressure. LDF: laser Doppler flowmetry.

Cortical cerebral blood flow (CBF) can be monitored with laser Doppler flowmetry, and CBF plotted against CPP shows the autoregulatory curve, called Lassen's curve, with clear lower limit of autoregulation (LLA) (Figure 3). However while this is illustrative of the principle, in clinical practice an indicator able to predict the autoregulatory reserve is needed, in order to demonstrate how far the patient is from the LLA.

A number of useful secondary indices are available for this purpose. One of these is the pressure reactivity index (PRx) [8]. It is calculated from slow fluctuation of the arterial blood pressure (ABP) of a period between 20 seconds and 3 minutes. The response of the ICP may be passive when the vascular bed is not reactive. PRx, calculated as moving correlation coefficient between ABP and ICP with the pulse wave filtered out, is positive. With an active vascular bed, a rise in ABP produces vasoconstriction and a decrease in ICP. A decrease in ABP produces vasodilatation and an increase in ICP. Therefore, normally positive PRx starts to be negative, indicating negative correlation between slow changes in ABP and ICP.

**Figure 4:**
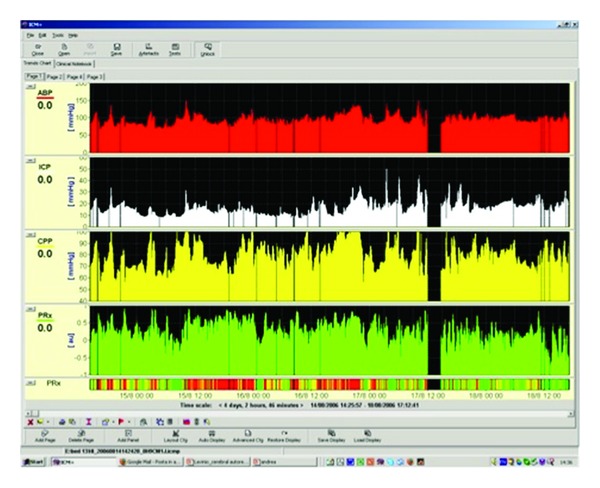
Example of multiple days monitoring of PRx along with ICP, ABP, and CPP where a patient attained good outcome after severe head injury. Image extrapolated from ICM+ software. ICP: intracranial pressure. ABP: arterial blood pressure. CPP: cerebral perfusion pressure. PRx: pressure reactivity index.

**Figure 5:**
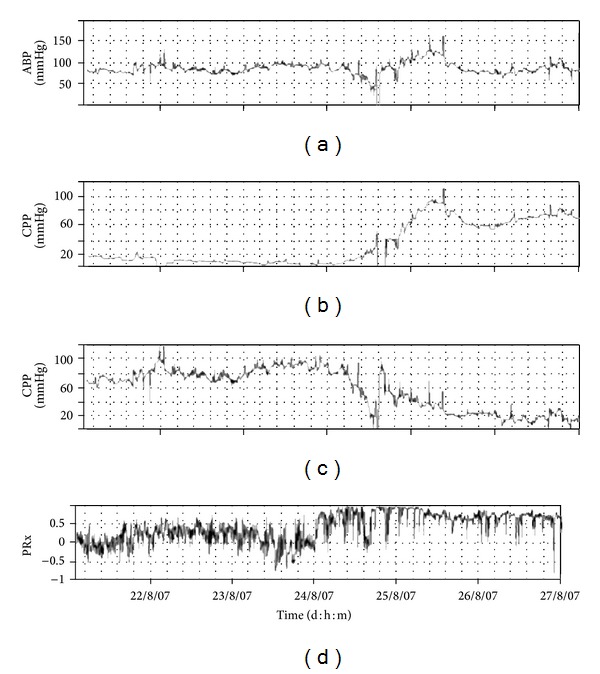
Increase in ICP above 20 and then above 80 mmHg. PRx in this case deteriorates almost half of a day before. ICP: intracranial pressure. ABP: arterial blood pressure. CPP: cerebral perfusion pressure. PRx: pressure reactivity index.

PRx is a “moving index,” able to be calculated continuously, forming a new variable.  Figure 4 shows an example of multiple days monitoring of PRx along with ICP, ABP, and CPP where a patient attained good outcome after severe head injury. It is possible to see that pressure reactivity may fluctuate in time after TBI. In cases of refractory intracranial hypertension, PRx sometimes deteriorates before the onset of intracranial hypertension.  Figure 5 shows an increase in ICP above 20 and then above 80 mmHg. PRx in this case deteriorates almost half of a day before.

**Figure 6:**
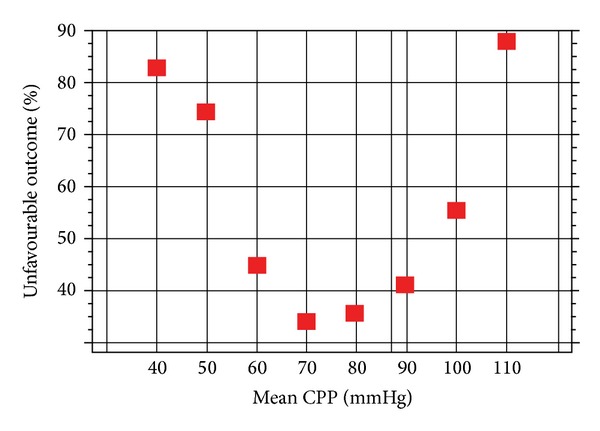
Mortality rate, expressed as a function of CPP, shows distinctive “U shape” curve. CPP: cerebral perfusion pressure.

PRx gives similar results to ICP when plotted against mortality rate, but with even steeper threshold. Mortality rate, expressed as a function of CPP, shows distinctive “U shape” curve (Figure 6). It indicates that the majority of patients who die have either too low or too high CPP. Whether what is “inadequate” CPP, is still a cause of debate. But if in the same cohort of patients PRx versus CPP is plotted, a similar “U shape” can be traced.

**Figure 7:**
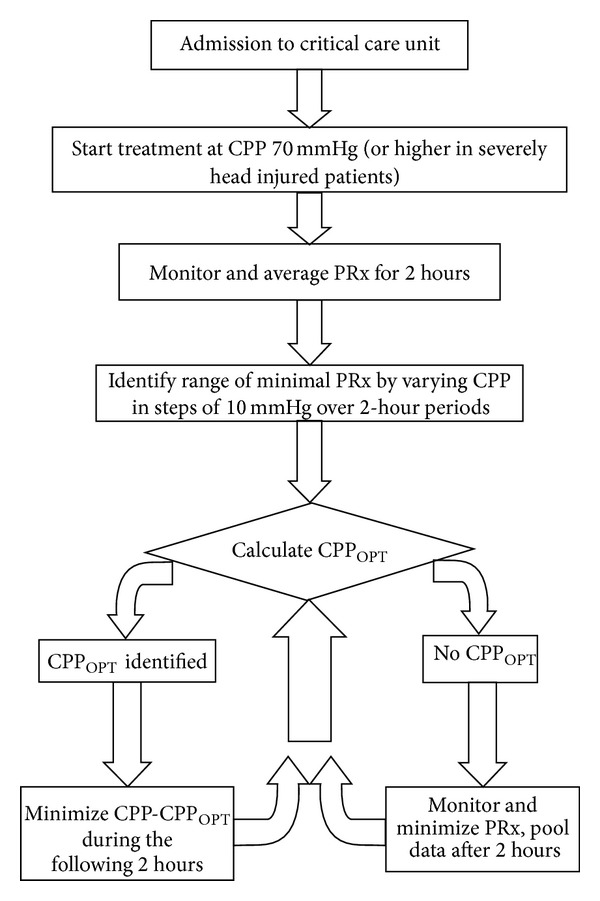
An algorithm to trace the PRx/CPP curve. CPP: cerebral perfusion pressure. PRx: pressure reactivity index.

An algorithm (Figure 7) was proposed by Steiner in order to trace the PRx/CPP curve [11]. The minimum of the curve was named “optimal CPP.” Steiner, using retrospective data, proved that greater distance between “optimal” and current CPP associates with worse outcome following TBI. A randomized trial is required to see whether “optimization” of CPP may improve outcome after TBI.

## 4. Brain Tissue Oxygenation (PbtO_2_)

The second most popular brain monitoring modality is brain tissue oxygenation (PbtO_2_). In order to monitor it, a thin electrode, able to detect oxygen content, is introduced into the brain parenchyma, allowing only a very small sampling area, just above a cubic millimetre.

Long-term trend (several days) of PbtO_2_ recorded along ICP, ABP, and CPP frequently fails to show any association between the modalities. However if selected time periods with more dynamical changes in PbtO_2_ are used, the positive correlation between CPP and PbtO_2_ becomes more apparent [12]. This perhaps indicates that by increasing CPP it is possible to improve brain tissue oxygenation, and this is interpreted in such a way by many neuroscientists.

The main problem in PbtO_2_ is that it may change dramatically, regarding different regions of the brain. Work conducted in Cambridge by Gupta and colleagues shows good correlation between PbtO_2_ and jugular bulb blood oxygenation only in areas without focal injury. In areas with focal injury, the correlation is absent. This has also been confirmed in other studies [13].

Ingenious idea was proposed by Jaeger et al. [14] to use PbtO_2_ to calculate an oxygen reactivity index; in a similar way PRx is calculated, by correlating changes in PbtO_2_ and CPP from the time window of about 1 hour. Positive ORX was supposed to indicate the situation when PbtO_2_ may be improved by increasing CPP, and ORX around zero when no further oxygenation improvement would be achieved with increasing CPP. At least, in the range of positive ORX, this index should be associated to PRx, but unfortunately this cannot be confirmed by other studies [15].

## 5. Near Infrared Spectroscopy (NIRS) and Transcranial Doppler (TCD)

Near infrared spectroscopy (NIRS) is a noninvasive modality to monitor changes in oxygen saturation in the brain. Compared to the previously described modality, it covers several cubic centimetres when applied to a central lobe. The theoretical basis lies on the fact that in the context of intracranial hypertension, slow waves in ICP have been found, which occur in 0.3 to 2.0 cycles per minute. Several studies demonstrated a relationship between those slow waves with ICP and cerebral oxygenation [16].

**Figure 8:**
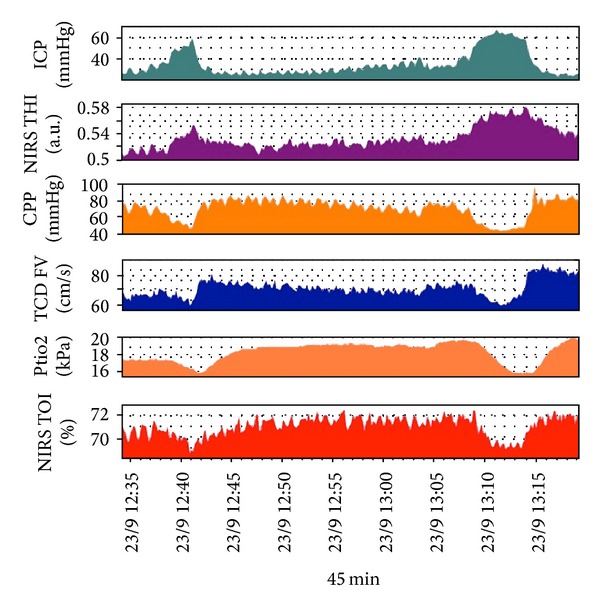
Multimodal monitoring during a plateau wave of ICP, demonstrating a decrease in CPP, PbtO_2_, blood flow velocity (which was assessed with TCD), and HbO_2_ during the curve, resulting in an ischaemic insult. ICP: intracranial pressure. NIRS THI: near infrared spectroscopy, tissue haemoglobin index. CPP: cerebral perfusion pressure. TCD FV: Trans Cranial Doppler Flow Velocity. PtiO2: brain tissue oxygenation. NIRS TOI: near infrared spectroscopy, tissue oxygenation index.

Figure 8 shows multimodal monitoring during a plateau wave of ICP, demonstrating a decrease in CPP, PbtO_2_, blood flow velocity (which was assessed with TCD), and HbO_2_ during the curve, resulting in an ischaemic insult.

NIRS can record relative changes in the concentration of deoxygenated (Hb) and oxygenated (HbO_2_) haemoglobin [16]. Normally, fluctuations in Hb and HbO_2_ are normally negatively correlated with each other, but in the presence of the vasogenic waves of ICP they would change in the same directions [16].

Experiments on arterial hypotension in piglets conducted at the John's Hopkins universty, revealed LLA, using laser Doppler and Somanetics cerebral oxygenation index [17]. In the same institution similarly good association between NIRS and TCD-divided indices was found in autoregulation indices derived with TCD and NIRS [18].

Positive association between the measurements—divided TOX index and TCD-assessed autoregulation—was documented in a group of patients with sepsis in the study from Basel [19].

## 6. Conclusion

In conclusion ICP still remains the most important modality of brain monitoring. However there is contrasting evidence that monitoring of ICP is associated with better outcome, and further prospective, multicentre, randomized trials, based on management protocol, are needed. Nobody can expect the outcome to improve by monitoring only, but it should be a monitoring-management combo instead.

PbtO_2_, apart from the initial enthusiasm, is not certain. The most significant disadvantage is that we are a hostage of catheter placement area.

“Optimal CPP” seems to be an important and promising idea, but yet to be confirmed by an RCT. It may be understood as a consensus between CPP-oriented therapy, where the CPP should be kept high, and Lund concept, where CPP can be kept low in order to reduce transcapillary water leak, lower oedema, and ICP. “Optimal CPP” says: not too low, not too high, just maintained to the needs of individual.

Finally, computer bedside calculation of “secondary parameters” may be helpful—again should be subject of more prospective clinical studies. Do we need a hole? Maybe, in the future, not always. Many laboratories work on noninvasive ICP, based on TCD and arterial pressure analyses. We hope that noninvasive ICP will become feasible in the future.

## References

[1] Lavinio A, Timofeev I, Nortje J (2007). Cerebrovascular reactivity during hypothermia and rewarming. *British Journal of Anaesthesia*.

[2] Kim DJ, Czosnyka Z, Keong N (2009). Index of cerebrospinal compensatory reserve in hydrocephalus. *Neurosurgery*.

[3] Kim DJ, Kasprowicz M, Carrera E (2009). The monitoring of relative changes in compartmental compliances of brain. *Physiological Measurement*.

[4] Guillaume J, Janny P (1951). Continuous intracranial manometry; importance of the method and first. *Revue Neurologique*.

[5] Lundberg N (1960). Continuous recording and control of ventricular fluid pressure in neurosurgical practice. *Acta Psychiatrica Scandinavica*.

[6] Czosnyka M, Guazzo E, Whitehouse M (1996). Significance of intracranial pressure waveform analysis after head injury. *Acta Neurochirurgica*.

[7] Sorrentino E, Diedler J, Kasprowicz M (2012). Critical thresholds for cerebrovascular reactivity after traumatic brain injury. *Neurocritical Care*.

[8] Balestreri M, Czosnyka M, Hutchinson P (2006). Impact of intracranial pressure and cerebral perfusion pressure on severe disability and mortality after head injury. *Neurocritical Care*.

[9] Chesnut RM, Temkin N, Carney N (2012). A trial of intracranial-pressure monitoring in traumatic brain injury. *New England Journal of Medicine*.

[10] Hutchinson PJ, Kolias AG, Czosnyka M, Kirkpatrick PJ, Pickard JD, Menon DK (2013). Intracranial pressure monitoring in severe traumatic brain injury. *British Medical Journal*.

[11] Steiner LA, Czosnyka M, Piechnik SK (2002). Continuous monitoring of cerebrovascular pressure reactivity allows determination of optimal cerebral perfusion pressure in patients with traumatic brain injury. *Critical Care Medicine*.

[12] Radolovich DK, Czosnyka M, Timofeev I (2010). Transient changes in brain tissue oxygen in response to modifications of cerebral perfusion pressure: an observational study. *Anesthesia and Analgesia*.

[13] Valadka AB, Gopinath SP, Contant CF, Uzura M, Robertson CS (1998). Relationship of brain tissue Po2 to outcome after severe head injury. *Critical Care Medicine*.

[14] Jaeger M, Schuhmann MU, Soehle M, Meixensberger J (2006). Continuous assessment of cerebrovascular autoregulation after traumatic brain injury using brain tissue oxygen pressure reactivity. *Critical Care Medicine*.

[15] Radolovich DK, Czosnyka M, Timofeev I (2009). Reactivity of brain tissue oxygen to change in cerebral perfusion pressure in head injured patients. *Neurocritical Care*.

[16] Weerakkody RA, Czosnyka M, Zweifel C (2012). Near infrared spectroscopy as possible non-invasive monitor of slow vasogenic ICP waves. *Acta Neurochirurgica*.

[17] Brady KM, Lee JK, Kibler KK, Easley RB, Koehler RC, Shaffner DH (2008). Continuous measurement of autoregulation by spontaneous fluctuations in cerebral perfusion pressure: comparison of 3 methods. *Stroke*.

[18] Brady K, Joshi B, Zweifel C (2010). Real-time continuous monitoring of cerebral blood flow autoregulation using near-infrared spectroscopy in patients undergoing cardiopulmonary bypass. *Stroke*.

[19] Steiner LA, Pfister D, Strebel SP, Radolovich D, Smielewski P, Czosnyka M (2009). Near-infrared spectroscopy can monitor dynamic cerebral autoregulation in adults. *Neurocritical Care*.

